# Association between Vascular Inflammation and Inflammation in Adipose Tissue, Spleen, and Bone Marrow in Patients with Psoriasis

**DOI:** 10.3390/life11040305

**Published:** 2021-04-01

**Authors:** Hannah Kaiser, Amanda Kvist-Hansen, Martin Krakauer, Peter Michael Gørtz, Kristoffer Mads Aaris Henningsen, Xing Wang, Christine Becker, Claus Zachariae, Lone Skov, Peter Riis Hansen

**Affiliations:** 1 Department of Cardiology, Herlev and Gentofte Hospital, 2900 Hellerup, Denmark; amanda.kvist-hansen@regionh.dk (A.K.-H.); kristoffer.mads.aaris.henningsen@regionh.dk (K.M.A.H.); peter.riis.hansen@regionh.dk (P.R.H.); 2 Department of Dermatology and Allergy, Herlev and Gentofte Hospital, 2900 Hellerup, Denmark; claus.zachariae@regionh.dk (C.Z.); lone.skov.02@regionh.dk (L.S.); 3 Department of Clinical Medicine, University of Copenhagen, 2200 Copenhagen, Denmark; 4 Department of Clinical Physiology and Nuclear Medicine, Herlev and Gentofte Hospital, 2900 Hellerup, Denmark; martin.krakauer@regionh.dk (M.K.); peter.michael.goertz.02@regionh.dk (P.M.G.); 5 Department of Clinical Physiology and Nuclear Medicine, Bispebjerg and Frederiksberg Hospital, 2400 Copenhagen, Denmark; 6 Department of Medicine, Division of Clinical Immunology, Icahn School of Medicine at Mount Sinai, New York, NY 10029, USA; xing.wang@mssm.edu (X.W.); christine.becker@mssm.edu (C.B.); 7 Department of Genetics and Genomic Sciences, Icahn School of Medicine at Mount Sinai, New York, NY 10029, USA

**Keywords:** psoriasis, cardiovascular disease, ^18^F-FDG-PET/CT, vascular inflammation, adipose tissue

## Abstract

Psoriasis is associated with atherosclerotic cardiovascular disease (CVD) with significant overlap of inflammatory pathways. A link between vascular inflammation and inflammation in multiple adipose tissue types, spleen, and bone marrow may exist. Therefore, we investigated these associations using ^18^F-fluorodeoxyglucose positron emission tomography/computed tomography (^18^F-FDG-PET/CT) in patients with psoriasis (*n* = 83) where half had established CVD. Carotid ultrasound imaging was also performed. Inflammation was measured by FDG uptake in the aorta, visceral- (VAT), subcutaneous- (SAT), and pericardial (PAT) adipose tissues, and spleen and bone marrow, respectively. Vascular inflammation was associated with FDG uptakes in all adipose tissues, including VAT (β = 0.26; *p* < 0.001), SAT (β = 0.28; *p* < 0.001), PAT (β = 0.24; *p* < 0.001), spleen (β = 1.35; *p* = 0.001), and bone marrow (β = 1.14; *p* < 0.001). Adjustments for age, sex, body mass index, and high sensitivity C-reactive protein did not change the results. These associations were generally preserved in the patients without prior CVD. No associations were observed between vascular inflammation and carotid intima-media thickness or presence of carotid plaques, respectively. The results suggest an inflammatory link between vascular and adipose tissues, spleen, and bone marrow in patients with psoriasis.

## 1. Introduction

Psoriasis is a chronic systemic inflammatory disease with many comorbidities, e.g., obesity and cardiovascular disease (CVD) [1]. Inflammation plays a critical role in atherosclerosis, the main contributor to CVD, and systemic low-grade inflammation is thought to be an essential link between the two diseases [2,3]. Indeed, in patients with psoriasis, ^18^F-fluorodeoxyglucose positron emission tomography/computed tomography (^18^F-FDG-PET/CT) studies have demonstrated increased inflammation determined by FDG uptake in the aorta and diverse other tissues, e.g., subcutaneous adipose tissue (SAT), spleen, liver, and bone marrow, and in these patients, vascular inflammation is linked with subclinical coronary artery disease [4,5,6,7,8]. Pericardial adipose tissue (PAT) is usually considered to include epicardial adipose tissue (EAT) beneath the visceral pericardial layer and the fat on the external surface of the parietal pericardial layer (paracardial fat), respectively, and EAT is of particular interest by representing a true visceral fat depot that surrounds the coronary arteries and increases proportionally with obesity and visceral adipose tissue (VAT) [9,10,11]. Notably, inflammation in this pericoronary fat depot may have “outside-in” paracrine inflammatory effects that contribute to coronary artery disease [9,10,11]. In patients with psoriasis, EAT volume is increased and linked to subclinical atherosclerosis, i.e., coronary artery calcification and increased carotid artery intima-media thickness (CIMT) [12,13,14], but whether this is associated with overall PAT inflammation is less clear. Additionally, there is limited systematic evidence available on associations between vascular inflammation, CIMT, and inflammation in selected tissues, e.g., spleen and bone marrow in these patients [4,5,6]. Therefore, we assessed inflammation determined by FDG uptake in the aorta, VAT, SAT, PAT, spleen, and bone marrow by ^18^F-FDG-PET/CT in patients with psoriasis and explored correlations between vascular inflammation and inflammation in the examined tissues, CIMT, and presence of carotid artery atherosclerotic plaques, respectively.

## 2. Materials and Methods

This study was conducted as a part of a large multi-scale study investigating the association between CVD and psoriasis (ethical committee ID H-17003458). Patients with plaque psoriasis aged ≥ 30 years where approximately half of the patients had prior atherosclerotic CVD (myocardial infarction, ischemic stroke, coronary revascularization, and/or peripheral artery disease) were examined with ^18^F-FDG-PET/CT. Psoriasis area and severity index (PASI) and body mass index (BMI) were measured and blood samples analyzed for lipid profile, glycated haemoglobin (HbA1c) and high-sensitivity C-reactive protein (hs-CRP).

**Figure 1:**
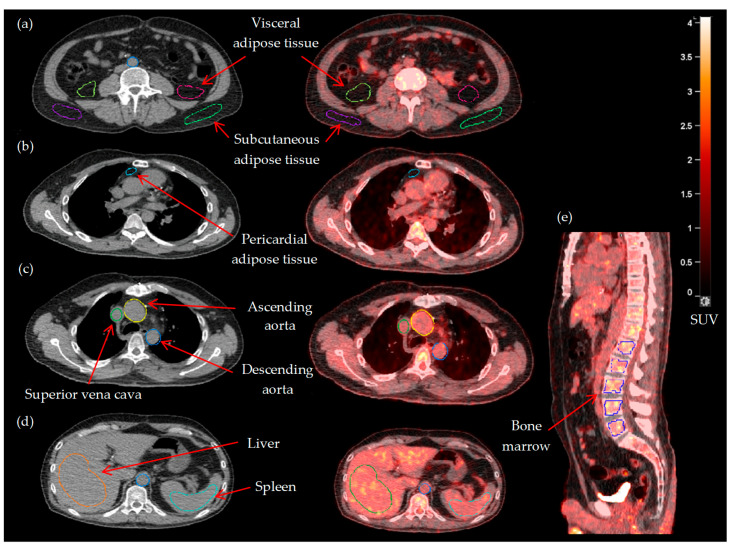
Transverse and coronal images from computed tomography (left) with corresponding positron emission tomography (right) with arrows indicating regions-of-interests (ROIs) drawn in (**a**) visceral and subcutaneous adipose tissue, (**b**) pericardial adipose tissue, (**c**) ascending and descending aorta and superior vena cava, (**d**) spleen and liver, and (**e**) bone marrow (lumbar vertebrae 1–5). SUV, standardized uptake value.

Patients received intravenous ^18^F-FDG (3.5 MBq/kg) 120 minutes prior to the FDG-PET/CT scan that was performed on a GE Discovery 710 scanner (General Electric Medical Systems, Milwaukee, WI, USA). An unenhanced low-dose CT scan was performed for anatomic localization and attenuation correction, and region-of-interests (ROIs) were manually delineated by using the MIM 6.9.2 software (Cleveland, OH, USA) (Figure 1). Arterial FDG uptake was quantified by using the average of maximum target-to-background (TBR_max_) values as a measure of vascular inflammation in accord with established methodology [15]. Three mm thick ROIs were placed encompassing both the arterial lumen and the wall on all axial slices of the aorta, and within each ROI, the maximum standardized uptake value (SUV_max_) of FDG was recorded as time- and dose-corrected tissue radioactivity divided by body weight. The blood-corrected TBR_max_ was quantified by dividing SUV_max_ of each slice of the arterial ROIs with SUV_mean_ of the reference activity in the superior vena cava (VCS), and the mean TBR_max_ from all arterial slices representing inflammation in the entire aorta was calculated. ROIs for VAT, SAT, and PAT were drawn in 3D encompassing as much as the target tissue as possible, avoiding edges and FDG spillover from neighboring organs and tissues. PAT was defined as the adipose tissue anterior to the pericardium at the level of the aortic root. Two symmetrical ROIs were placed in VAT caudal to the kidneys and SAT at the same transverse body level in the loin, respectively. SUV_mean_ for the respective adipose tissue was divided by the SUV_mean_ of VCS to produce the blood-corrected tissue TBR_mean_. FDG uptakes in the bone marrow (lumbar vertebrae 1–5) and spleen were quantified as average SUV_means,_ and the metabolic activities in the spleen and the bone marrow were reported as the spleen-to-liver ratio (SLR) and the bone marrow-to-liver ratio (BLR), respectively, by dividing respective SUV_means_ with the SUV_mean_ of the liver [6,16].

CIMT was measured using an Affiniti 70G ultrasound system with a 5–12 MHz linear array transducer (Philips Ultrasound Inc., Bothell, WA, USA). The right and the left CIMTs were measured on a 10 mm far wall segment of the distal common carotid artery during diastole according to the Mannheim consensus [17]. Both carotid arteries were also visualized for presence of atherosclerotic plaques.

Linear regression analysis was used to investigate associations between ^18^F-FDG-PET/CT-determined vascular inflammation in the entire aorta and inflammation in the different examined tissues (PAT, VAT, SAT, spleen, and bone marrow). Associations between FDG uptakes and CIMT and presence of carotid artery plaques were also examined. In addition, Spearman correlation analysis was used for determination of the association between vascular inflammation and PASI. Multivariable regression analysis was used in order to adjust for age, sex, BMI, and hs-CRP. Model assumptions were applied for all regression models by using residual-plots. Log_2_-transformation of the models with adipose tissues (VAT, SAT, and PAT) showed better model assumptions and were therefore used in the analyses. Logistic regression was used for carotid plaques as a binary outcome (no plaques or plaques in one/both carotid arteries) with FDG uptake in the entire aorta as the exposure. Statistical analysis was performed using R, version 3.6.1 (R Foundation for Statistical Computing, Vienna, Austria).

## 3. Results

The study comprised a total of 83 patients with moderate to severe psoriasis of whom 39 (47.0%) had prior CVD (Table 1). Subjects were mainly men (*n* = 60, 72.3%) with mean (SD) age 59.6 (10.9) years and BMI 30.0 (5.6) kg/m^2^. Nearly one-quarter of patients also had psoriatic arthritis (*n* = 20, 24.1%) and/or diabetes (*n* = 20, 24.1%), and 53.0% (*n* = 44) were currently receiving systemic antipsoriatic treatment.

**Figure 2:**
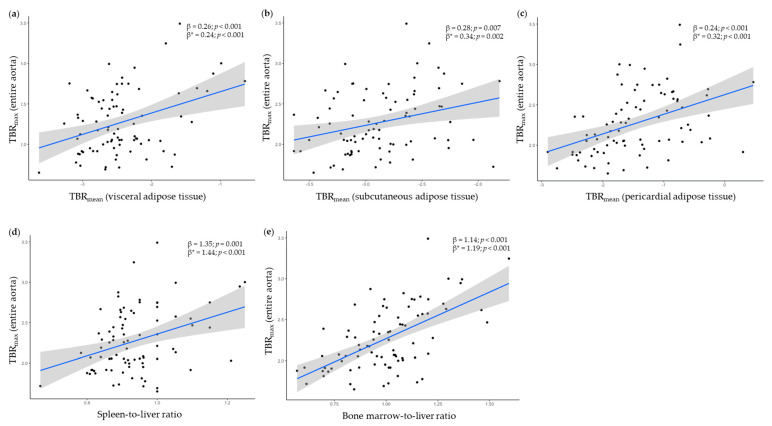
Regression plots of associations between ^18^F-fluorodeoxyglucose (FDG) uptake in the entire aorta and FDG uptake in (**a**) visceral-, (**b**) subcutaneous-, and (**c**) pericardial adipose tissue, (**d**) spleen-to-liver ratio, and (**e**) bone marrow-to-liver ratio in all patients with psoriasis (*n* = 83) examined by ^18^F-fluorodeoxyglucose positron emission tomography/computed tomography. Regression coefficients; β = unadjusted, β* = adjusted for age, sex, body mass index, and high-sensitivity C-reactive protein. Shaded regions represent 95% confidence intervals for the fitted regression models. Visceral-, subcutaneous-, and pericardial adipose tissue values were log_2_transformed. TBR: target-to-background ratio.

Analyses of the relationship between vascular inflammation in the entire aorta and FDG uptakes in the various adipose tissues, spleen, and bone marrow are shown in Table 2 and Figure 2. A significant positive association was found between vascular inflammation and FDG uptakes in all adipose tissues including VAT (β = 0.26; *p* < 0.001), SAT (β = 0.28; *p* < 0.001), and PAT (β = 0.24; *p* < 0.001). These associations remained significant after adjustment for age, sex, BMI, and hs-CRP (Table 2 and Figure 2). In addition, there were significant associations between vascular inflammation and FDG uptakes in the spleen (SLR, β = 1.35; *p* = 0.001) and the bone marrow (BLR, β = 1.14; *p* < 0.001). These associations also remained significant after adjustment. Analyses limited to patients without CVD (*n* = 44) showed similar significant positive associations between vascular inflammation and FDG uptakes without/with adjustment for age, sex, BMI, and hs-CRP for VAT (β = 0.29; *p* = 0.002/β = 0.27; *p* = 0.011), SAT (β = 0.39; *p* = 0.010/β = 0.42; *p* = 0.011), PAT (β = 0.32; *p* < 0.001/β = 0.40; *p* < 0.001), and bone marrow (BLR, β = 1.24; *p* < 0.001/β = 1.36; *p* < 0.001) but not for the spleen (SLR, β = 0.49; *p* = 0.491/β = 0.86; *p* = 0.262) (Table 2).

No association between vascular inflammation and CIMT was observed in unadjusted (β = −0.48; *p* = 0.164) or adjusted (β = −0.15; *p* = 0.680) analyses in the overall cohort. In addition, logistic regression analysis showed no significant association between vascular inflammation and presence of atherosclerotic plaques in the carotid arteries (unadjusted odds ratio (OR) 0.33 [95% CI 0.10–1.03]; *p* = 0.062 and adjusted OR 0.46 (95% CI 0.10–1.93); *p* = 0.286) and no association was observed between vascular inflammation and PASI in patients without systemic antipsoriatic treatment (*n* = 39) with PASI median (IQR) 11.0 (6.6–14.3) (r = −0.10; *p* = 0.561).

## 4. Discussion

This study found an association between vascular inflammation and FDG uptake in adipose tissues including VAT, SAT, PAT, spleen, and bone marrow, respectively, in patients with psoriasis examined by ^18^F-FDG-PET/CT. These associations remained significant after adjustment for age, sex, BMI and hs-CRP. No association was found between vascular inflammation and CIMT, atherosclerotic plaques in the carotid arteries, and PASI, respectively.

Although we are not aware of studies reporting FDG uptakes collected from all the currently examined tissues in the same group of individuals, some of our results are consistent with those of previous studies showing an association between vascular inflammation and FDG uptake in SAT, spleen, and bone marrow in patients with psoriasis [5,6,7]. The latter results add to emerging evidence linking increased metabolic activity in the spleen and the bone marrow representing increased hematopoietic stress, inflammatory cell differentiation, and extramedullary hematopoiesis to inflammatory diseases such as atherosclerosis, and it is notable that increased splenic metabolic activity appears to be an independent predictor of recurrent CVD [18,19]. In addition, while it was previously observed that arterial and fat tissue inflammation are highly correlated in subjects with prior atherosclerotic CVD or multiple traditional CVD risk factors [20], the present study is, to our knowledge, the first to demonstrate an association between vascular inflammation and FDG uptake in VAT and PAT in patients with psoriasis.

Vascular inflammation determined by ^18^F-FDG-PET/CT is increased in patients with psoriasis and is prognostic for future CVD [5,21]. While FDG uptake in larger arteries including the aorta and the carotid arteries has been linked with vessel wall inflammation, e.g., infiltration of macrophages and presence of atherosclerotic plaques, mechanisms underlying FDG uptake in other tissues and clinical conditions may be less well defined [22,23]. However, increased FDG uptake is generally viewed as a marker of tissue inflammation, and the present demonstration of an association between vascular inflammation and inflammation in diverse other examined tissues clearly supports the established view of psoriasis as a systemic inflammatory disease [1,2]. Specifically, although EAT is usually considered to be a part of PAT, it is a unique extension of visceral fat that exerts paracrine effects on coronary arteries and can be a source of inflammatory mediators contributing to inflammation in the adjacent coronary arteries wall and, ultimately, coronary artery disease [9,10,11]. Indeed, inflammation in pericoronary EAT assessed by a fat attenuation index determined by coronary CT angiography was recently shown to be an independent risk factor for coronary artery disease [24,25]. EAT determined by echocardiography and CT is increased in patients with psoriasis and associated with coronary artery calcification and increased CIMT [12,13,14], but the link between increased EAT tissue volume and EAT inflammation determined by routine ^18^F-FDG-PET/CT is less clear. We found that vascular inflammation in patients with psoriasis was associated with FDG uptake in PAT, and although the precise contribution of EAT to FDG uptake in PAT as determined by current ^18^F-FDG-PET/CT techniques remains to be determined, the results appear to corroborate that PAT inflammation is of mechanistic importance for the risk of coronary artery disease in patients with psoriasis [1,2,3,9,10,11,24,25]. Along this line, systemic biologic antipsoriatic therapy was recently associated with reduced pericoronary EAT inflammation assessed by coronary CT angiography-determined perivascular fat attenuation index in patients with psoriasis [26]. We also found that the link between vascular inflammation and FDG uptake in PAT and other fat tissues was preserved in patients without CVD, supporting a general inflammatory cross-talk between fat tissues and arteries irrespective of established CVD and in potential accord with studies indicating that patients with psoriasis harbor significant subclinical atherosclerotic CVD [20,27]. Reports of EAT and/or PAT inflammation examined by ^18^F-FDG-PET/CT are scarce in the literature, but increased EAT inflammation has also been observed in patients with atrial fibrillation which may provide a mechanistic link to increased risk of atrial fibrillation observed in patients with psoriasis [28,29].

Associations between vascular inflammation and CIMT or presence of atherosclerotic plaques in the carotid arteries were not found in our study. We hypothesized such association since all these variables are viewed as markers of subclinical atherosclerosis. However, they represent pathophysiologically diverse vascular phenotypes and do not, for example, necessarily express the same state of vascular inflammation [30,31,32]. In addition, many patients in our study received statins for primary or secondary CVD prevention, which has been linked with decreased vascular inflammation and CIMT [33,34,35]. Therefore, effects of statins may have contributed to disruption of any potential association between these markers. We also did not find an association between vascular inflammation and PASI in patients without systemic antipsoriatic treatment, which is consistent with results of another study including patients with moderate-to-severe psoriasis without biologic antipsoriatic therapy [5]. However, such association has been reported by others, including concomitant reduction of vascular inflammation and PASI in patients receiving biologic antipsoriatic therapy and reasons for these differing results warrant further study [36,37].

The main limitations of the study include a relatively small sample size and no healthy comparable control group. The lack of a control group prevents conclusions on the role of psoriasis per se, and it is notable that associations between vascular inflammation and FDG uptake in adipose tissues have also been observed in patients without psoriasis with CVD or CVD risk factors [20]. In addition, the sample size does not allow for extensive adjustment for potential confounders. Moreover, our study included both patients with and without systemic antipsoriatic treatment, representing a cohort with different disease activity. Further limitations include methodological considerations regarding determination of FDG uptake as a marker of inflammation in examined tissues. In contrast to the accepted methodology for quantification of vascular inflammation by FDG uptake [15], there is no established consensus regarding the preferred ^18^F-FDG-PET/CT methodology for measurement of inflammation in other tissues such as adipose tissues. For example, differences of FDG circulation times before scan acquisition, anatomical localization of ROIs, and selection of PET variables (SUV or TBR) influence the results. Additionally, measuring FDG uptake in PAT and EAT using routine ^18^F-FDG-PET/CT is challenging, e.g., due to risk of FDG activity spillover from the myocardium and large vessels in addition to difficulties differentiating between EAT and PAT because of movement artifacts and limited spatial resolution capacity of PET scans [10]. 

In conclusion, this study showed that, in patients with psoriasis, vascular inflammation is linked with inflammation in adipose tissues, spleen, and bone marrow. Although this association may exist in subjects without psoriasis as well, the results may underline that psoriasis is a systemic inflammatory disease, and more studies of the inflammatory crosstalk between arteries and adipose and hematopoietic tissues are warranted.

## Figures and Tables

**Table 1:** Clinical characteristics, laboratory measurements and results of ^18^F-fluorodeoxyglucose positron emission tomography/computed tomography and ultrasound imaging of carotid arteries in patients with psoriasis (*n* = 83).

	All Patients (*n* = 83)
Sex, Male, *n* (%) Age (years)	60 (72.3) 59.6 ± 10.9
BMI (kg/m^2^)	30.0 ± 5.6
Waist-to-hip ratio	0.99 ± 0.07
PASI	3.5 (1.1–10.8)
PsA, *n* (%)	20 (24.1)
Medically treated hypertension, *n* (%)	36 (43.4)
Medically treated diabetes, *n* (%)	20 (24.1)
Systemic antipsoriatic treatment, *n* (%)	44 (53.0)
Statin treatment, *n* (%)	41 (49.4)
Prior CVD, *n* (%)	39 (47.0)
Smoking, present or previous, *n* (%)	61 (73.5)
HbA1c (mmol/mol)	36.0 (34.0–41.0)
Total cholesterol (mmol/L)	4.43 ± 0.98
LDL-C (mmol/L)	2.39 ± 0.84
HDL-C (mmol/L)	1.24 ± 0.38
Triglycerides (mmol/L)	1.87 ± 0.97
Blood glucose (mmol/L)	5.9 (5.4–6.7)
hs-CRP (mg/L)	1.41 (0.68–3.83)
Entire aorta (TBR_max_)	2.27 ± 0.39
Visceral adipose tissue (TBR_mean_)	0.17 (0.15–0.21)
Subcutaneous adipose tissue (TBR_mean_)	0.13 (0.11–0.16)
Pericardial adipose tissue (TBR_mean_)	0.34 (0.26–0.52)
Spleen (SLR_mean_)	0.94 ± 0.10
Bone marrow (BLR_mean_)	1.00 ± 0.20
Carotid intima-media thickness (mm)	0.73 ± 0.13
Carotid atherosclerotic plaques, *n* (%)	50 (60.2)

Parametric data are reported as mean ± SD and non-parametric data are median (IQR). BMI, body mass index; PASI, psoriasis severity area index; PsA, psoriatic arthritis, CVD, cardiovascular disease; Hba1c, glycated haemoglobin; LDL-C, low-density lipoprotein cholesterol; HDL-C, high-density lipoprotein cholesterol; hs-CRP; high-sensitivity C-reactive protein; TBR, target-to-background; SLR, spleen-to-liver ratio; BLR, bone marrow-to-liver ratio.

**Table 2:** Analyses of the association between vascular inflammation and visceral-, subcutaneous-, and pericardial adipose tissue, spleen, and bone marrow in all patients with psoriasis and limited to patients without prior cardiovascular disease (CVD).

	All Patients (*n* = 83)	No CVD (*n* = 44)
	β (*p*-Value)	β (*p*-Value)
**Visceral adipose tissue ^1^** Unadjusted Adjusted	0.26 (<0.001) 0.24 (0.001)	0.29 (0.002) 0.27 (0.011)
**Subcutaneous adipose tissue ^1^** Unadjusted Adjusted	0.28 (0.007) 0.34 (0.002)	0.39 (0.010) 0.42 (0.011)
**Pericardial adipose tissue ^1^** Unadjusted Adjusted	0.24 (<0.001) 0.32 (<0.001)	0.32 (<0.001) 0.40 (<0.001)
**Spleen-to-liver ratio** Unadjusted Adjusted	1.35 (0.001) 1.44 (< 0.001)	0.49 (0.491) 0.86 (0.262)
**Bone marrow-to-liver ratio** Unadjusted Adjusted	1.14 (<0.001) 1.19 (<0.001)	1.24 (<0.001) 1.36 (<0.001)

Regression coefficients (β) from linear and multivariable regression models. ^1^ log_2_-transformed. Adjusted for age, sex, body mass index and high-sensitivity C-reactive protein.

## Data Availability

The data presented in this study are available on request from the corresponding author.
